# Low-reflective wire-grid polariser sheet in the visible region fabricated by a nanoprinting process

**DOI:** 10.1038/s41598-021-81750-2

**Published:** 2021-01-22

**Authors:** Ryohei Hokari, Kyohei Takakuwa, Hirohisa Kato, Akitaka Yamamoto, Yusuke Yamaguchi, Kazuma Kurihara

**Affiliations:** 1 Advanced Manufacturing Research Institute, National Institute of Advanced Industrial Science and Technology (AIST), AIST Tsukuba East, 1-2-1 Namiki, Tsukuba, Ibaraki 305-8564 Japan; 2 Ryoko Chemical Co., Ltd., PMO Nihonbashihoncho Bldg., 4-12-20, Nihonbashihon-cho, Chuo-ku, Tokyo, 103-0023 Japan; 3 Itoh Optical Industrial Co., Ltd., 3-19 Miyanari-cho, Gamagori, Aichi 443-0041 Japan; 4 Tokai Seimitsu Industrial Co., Ltd., 3-2-8 Honohara, Toyokawa, Aichi 442-0061 Japan

**Keywords:** Optical materials and structures, Surface patterning, Nanoparticles, Structural properties

## Abstract

For the construction of next-generation optical products and systems, the evolution of polariser sheets is a necessary requirement. To this end, a low-reflective wire-grid polariser (WGP) sheet for the visible light region is demonstrated, the nanowires of which consist of a sintered body of silver nanoparticle ink. The nanowires are formed by a nanoprinting process using a thermal nanoimprint method and ink filling. This process makes it easier to achieve multiple wafer-scale productions without using sophisticated equipment compared to conventional WGP nanofabrication techniques, which typically employ lithography and elaborate etching processes. The optical characteristics are controlled by the shape of the printed nanowires. A WGP sheet with a luminous degree of polarisation of 99.0%, a total luminous transmittance of 13.6%, and a luminous reflectance of 3.6% is produced. Its low reflectance is achieved through the uneven surface derived from the sintered body of the nanoparticle ink, and the shape of the bottom of the nanowire is derived from the tip shape of the mould structure. Furthermore, the printed WGP sheet has the durability required for the manufacturing of curved products, including sunglasses. The optical structures made of nanoparticle ink using this nanoprinting process have the potential to significantly contribute to the development of fine-structured optical elements with unprecedented functionality.

## Introduction

Polarisers are essential optical elements that support vital polarisation control technologies in a wide range of fields such as liquid crystal displays (LCDs), polarised sunglasses^1^, liquid crystal (LC) projectors, and in-vehicle head-up displays^2^. In-vehicle devices and LC projectors are used in high-temperature, high-humidity environments, and/or environments where there is prolonged exposure to high-intensity light, so the polarisers that make up these products are required to have high durability (high-temperature resistance, high-humidity resistance, and high-intensity light resistance). In contrast, in optical systems such as LC projectors, suppression of stray light remains a problem, and polarisers with low reflectance are required. Similarly, in polarised sunglasses, a polariser having high durability and low reflectance is required in order to suppress a change in colour and to prevent lens reflections. However, there are no thin polarisers that exhibit both high durability and low reflectance. If such a polariser could be realised, it would be possible to expand into new fields in which polariser-based applications have been difficult, and it would be expected to open up new possibilities for the technology.

Polarisers are roughly classified into dye-type polarisers^3,4^, WGPs^5–14^, and polarisers using a birefringent crystal such as calcite. Since these polarisers have different functioning principles, they have unique characteristics and are used appropriately according to their optical applications. WGPs used in the visible region of the electromagnetic spectrum have a grid structure of fine metal wires with a pitch shorter than the wavelength of the incident electromagnetic wave. They are manufactured using a micromachining process including physical vapour deposition, lithography, and etching processes. To obtain high polarisation performance and high transmittance, a dense and highly conductive metal structure with low optical absorption is used. Therefore, the incident polarised light on the light-blocking axis is reflected with a high reflectance much like when entering a metal mirror surface. For example, an aluminium WGP has a reflectance of ~ 80% for the incident polarised light on the light-blocking axis^15^. Since the structure providing their optical function is metal, they show excellent resistance to high temperature and high humidity as compared with dye-type polarisers. In recent years manufacturing methods combining nanoimprint and deposition methods have been developed^11–13^. Although the manufacturing cost has reduced compared to those manufactured by electron-beam lithography, it is still higher than that of dye-type polarisers. In a WGP for the visible light region, a thickness of several hundred nanometres is sufficient, which means that it can be incorporated into a product as a thin polariser sheet.

In this study, we propose an approach to reduce the reflectivity of WGPs to realise a highly durable polariser sheet with low reflectivity. Although various approaches have been researched and developed to reduce the reflectivity of WGPs, many of these methods involve laminating a light-absorbing material onto a wire-grid structure^15–17^, or using low-reflectivity metal on a wire-grid structure^18,19^. A moth-eye structure has been demonstrated as a method of suppressing the reflectance of a material surface^20–22^. This is effective not only for the dielectric transparent materials but also for the opaque and highly reflective surfaces of semiconductors and metals^20,23–27^. If such a moth-eye-like structure could be formed on the top and bottom surfaces of wire-grid structures, the reflectivity of a WGP sheet would be suppressed. However, it is difficult to form a moth-eye-like structure on the surface of the nanowire of a WGP for the visible light region using conventional processing methods, and it has not been realised until now. We focused on a printing technique that uses metal nanoparticle ink to form metal nanowires with irregularities on the surface. Compared to a metal structure formed using a physical vapour deposition process, the sintered body of a metal nanoparticle ink has an uneven surface depending on the sintering conditions^28^. However, since the nanowire structure of a WGP requires a pitch smaller than the wavelength of the target electromagnetic wave, it has been difficult to print and form the nanowire structure of the WGP for the visible light region. Instead, we have developed a nanoprinting process that forms thick ink patterns with a width of less than 100 nm^29–31^. This process involves filling nanoparticle ink into nanogrooves formed on a substrate surface using a nanoimprint method so that a nanowire structure having the required thickness for a WGP can be formed. In our study, a WGP sheet using the sintered body of a metal nanoparticle ink pattern was demonstrated. Furthermore, the optical properties of WGP sheets produced using moulds having different shapes were evaluated and compared, and the effect of reduction in the reflectance was confirmed.

## Results and discussion

**Figure 1:**
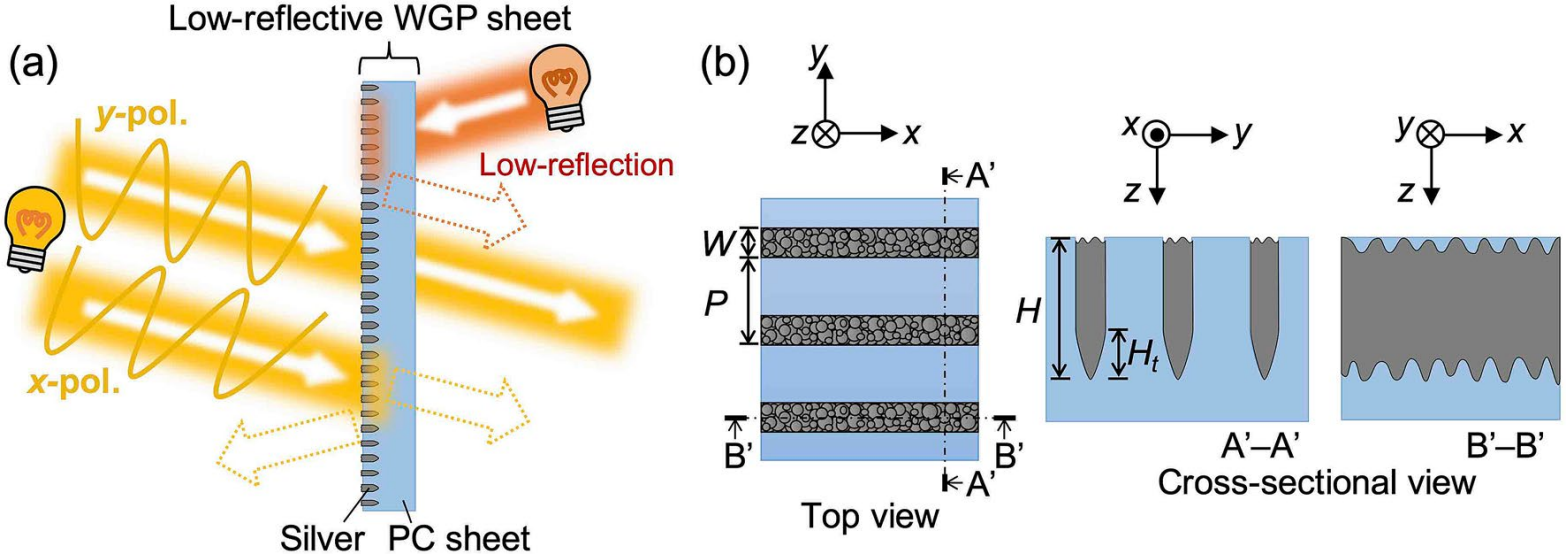
(**a**) Schematic of the low-reflective WGP sheet. (**b**) Schematics of the top and cross-sectional views of the surface structure.

Figure 1a shows a schematic of a low-reflectance WGP sheet, where the material of the metal nanowire is silver, and the substrate sheet is polycarbonate (PC). Figure 1b shows the geometry of the nanowire structure for a low-reflectivity WGP, where, *W*, *P*, *H*, and *H*_*t*_ represent the width, period, thickness of the nanowire, and the thickness of the tapered portion (uneven portion), respectively. The surface of the nanowire structure seen from the top side has irregularities (a moth-eye-like surface). The surface of the nanowire structure seen from the bottom side also has irregularities as well as a tapered shape.

To obtain a structure with the aforementioned characteristics where the silver nanoparticle ink could be embedded, the tip of the mould structure was processed so that it had a tapered shape with irregularities. Since the nanoprinting process allows the silver nanoparticle ink to be filled according to the shape of the inner part of the groove, the structure of the silver nanowire after sintering becomes practically the same shape, and irregularities are formed on the surface. The thickness of this uneven (tapered) part is important for reducing reflectance. Table 1 shows the geometric parameters of the four moulds used in this experiment. These values are the average of the values measured by scanning electron microscopy (SEM). *P* was designed to be fixed at 140 nm. The fabricated type-A mould had a width to pitch ratio of 37%, an *H*/*W* of 3.5, and an *H*_*t*_/*W* of 1.0. The type-C mould was fabricated to have a higher *H*/*W* ratio and more than twice the *H*_*t*_/*W* compared to the type-A and type-B moulds. The type-D mould, on the other hand, was fabricated to have a width to pitch ratio of 17%, a higher *H*/*W* of 15.1, and a higher *H*_*t*_/*W* of 7.5.

**Table 1:** Geometrical parameters of the moulds.

Mould type	*W* [nm]	*H* [nm]	*H*_*t*_ [nm]	*H*/*W*	*H*_*t*_/*W*
A	52.4	183	50	3.5	1.0
B	44.9	278	40	6.2	0.9
C	42.1	500	100	11.9	2.4
D	24.0	363	180	15.1	7.5

**Figure 2:**
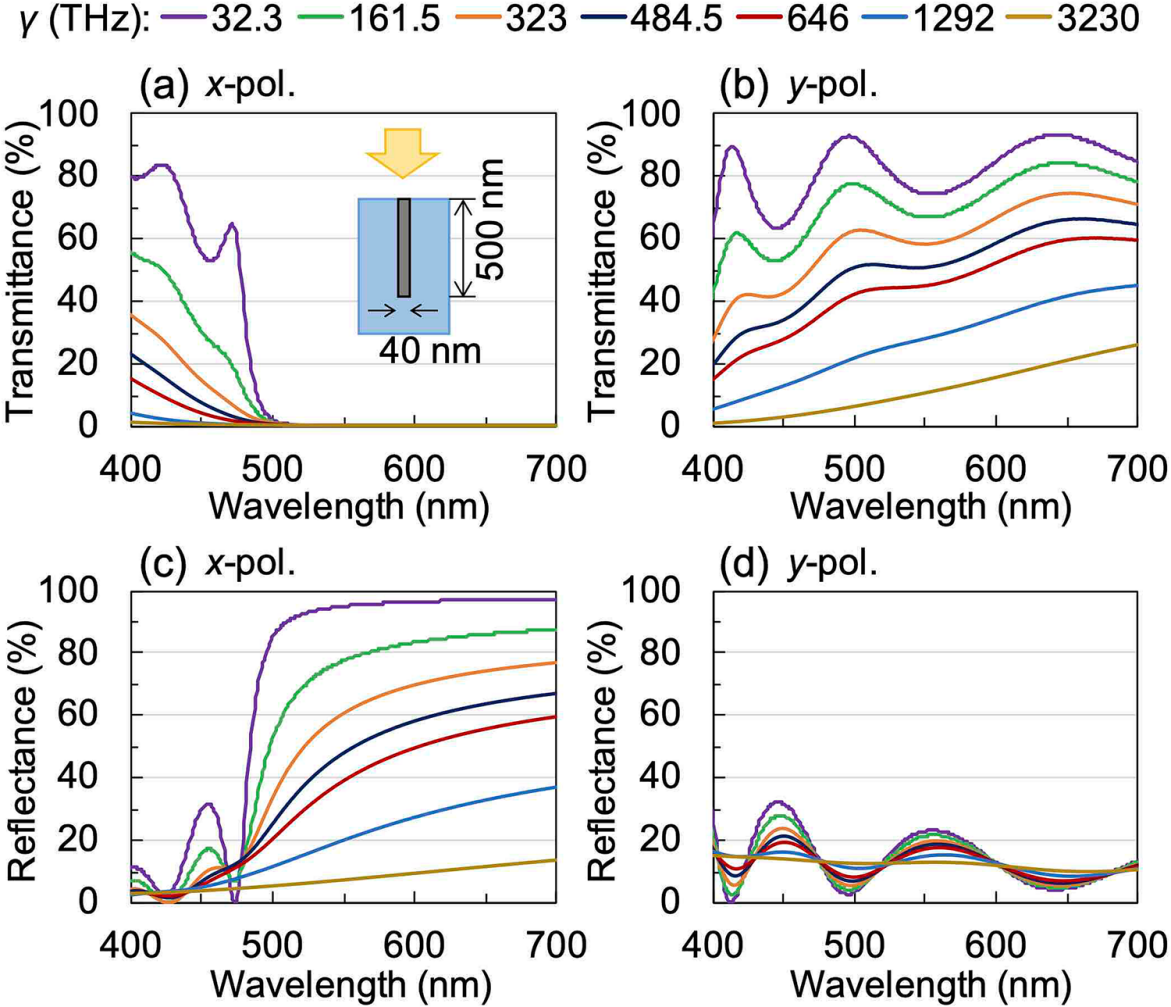
Calculated (**a**), (**b**) transmittance and (**c**), (**d**) reflectance spectra of embedded WGP sheet as a function of damping or collision frequency *γ*. The polarisation directions are (**a**), (**c**) *x*-polarisation and (**b**), (**d**) *y*-polarisation.

The optical characteristics of the WGP were calculated using a rigorous coupled-wave analysis (RCWA) simulation that predicts exact solutions for periodic structures based on Maxwell’s equations^32^. The effect of the main parameters that determine the optical characteristics of WGP was investigated. The refractive index and extinction coefficient of the PC substrate was set to 1.59 and 0 at wavelengths from 400 to 700 nm, respectively. The Drude model was used to calculate the dielectric constant of bulk silver, with the plasma frequency (*ω*_*p*_) and the damping or collision frequency (*γ*) set at 14.0 PHz and 32.3 THz, respectively^33,34^. It is important to note here that the dielectric constant of a sintered body of silver nanoparticle ink is different from that of bulk silver because of their different conductivities. The conductivity (*σ*) of metal in the visible region of the electromagnetic spectrum is represented by

$$\sigma \left( \omega \right) = \frac{{\varepsilon_{0} \omega_{p}^{2} }}{{\omega^{2} + \gamma^{2} }}\left( {\gamma + i\omega } \right),$$

where ε_0_ is the permittivity of the vacuum. Since the conductivity of a sintered body of silver nanoparticle ink varies depending on the sintering conditions^35^, the plasma frequency and the damping frequency also change. First, we investigated the effects resulting from the change in the damping frequency. Figure 2 shows the calculated transmittance and reflectance spectra of an embedded WGP sheet as a function of *γ* which was varied from 32.3 to 3230 THz. The inset in Fig. 2a illustrates the cross-sectional view of the model employed in our calculations. The model consists of a silver wire-grid structure (width 40 nm, thickness 500 nm, and pitch 140 nm) embedded in a PC substrate. We observed that both the transmittance and reflectance decreased with increasing *γ*.

**Figure 3:**
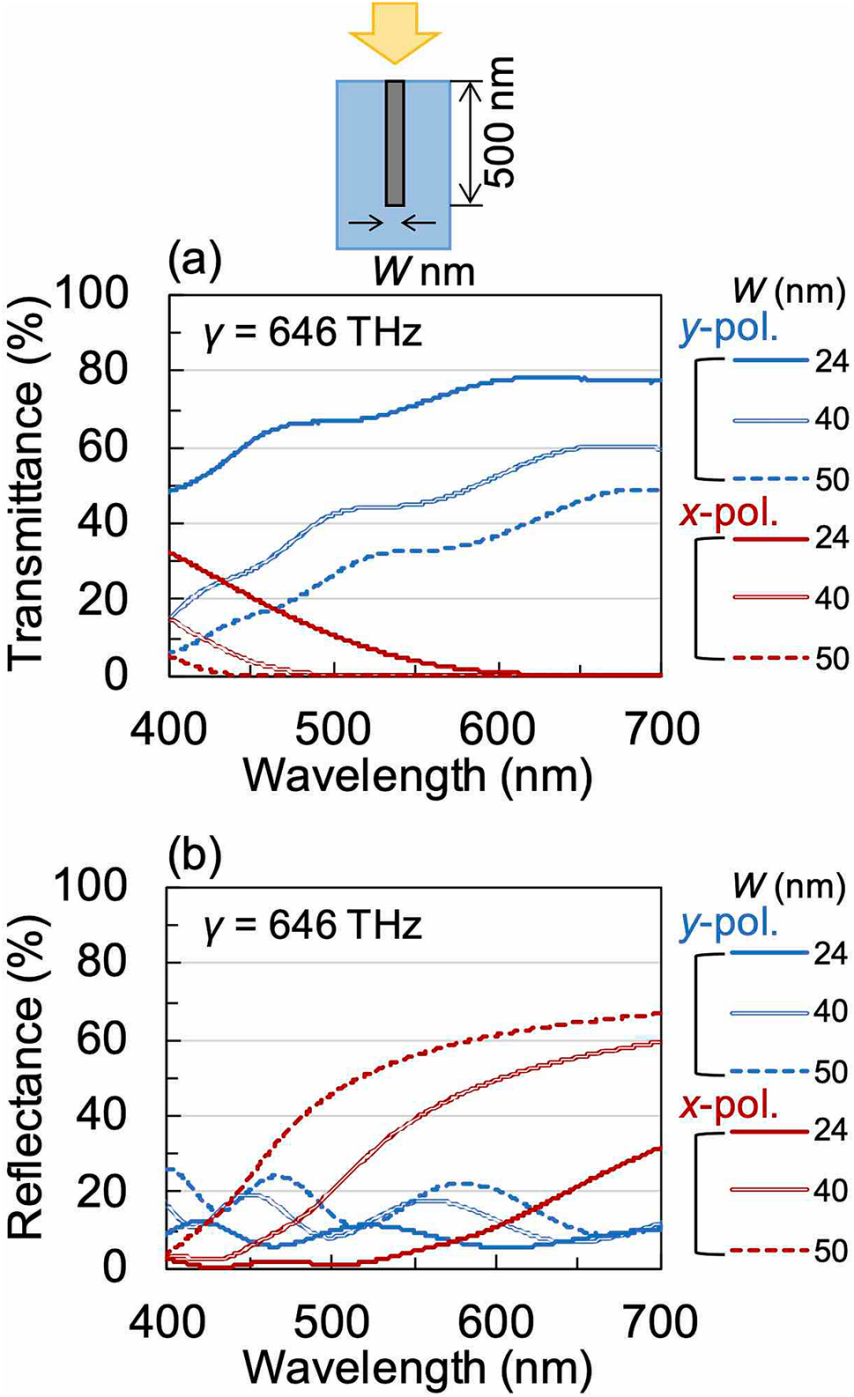
Calculated (**a**) transmittance and (**b**) reflectance spectra of embedded WGP sheet as a function of the width of the wire grid structure *W*.

**Figure 4:**
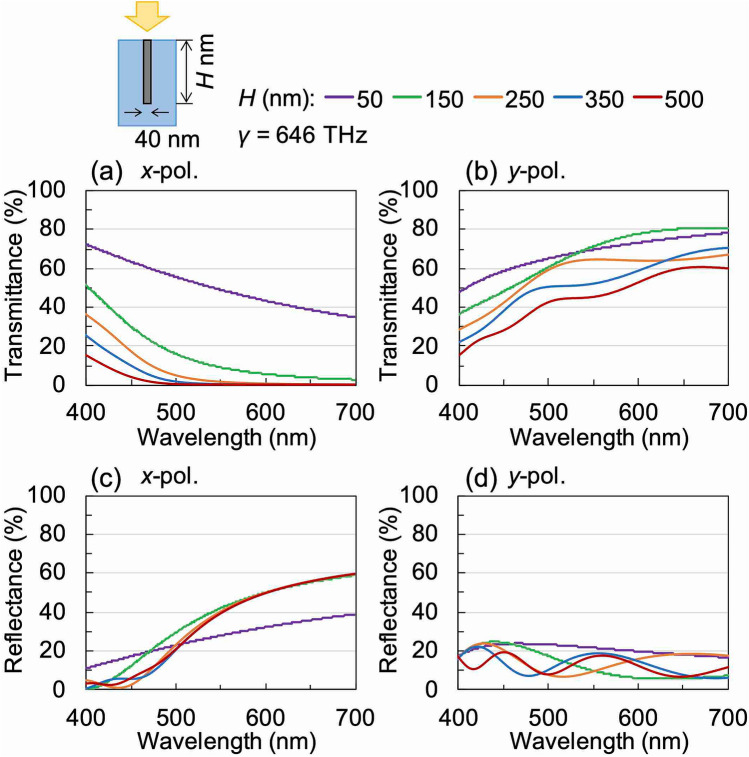
Calculated (**a**), *b) transmittance and **c**, **d** reflectance spectra of embedded WGP sheet as a function of the thickness of the wire grid structure *H*. The polarisation directions are *a), (**c**) *x*-polarisation and (**b**), (**d**) *y*-polarisation.

Next, using RCWA we investigated how the variation in the *W* and *H* of the wire grid structure influences the optical characteristics of WGP sheets fabricated from four moulds with different structures. In all calculations, *γ* was set to 646 THz, which was determined as an appropriate value by comparison with the experimental results described later. Figure 3 shows the calculated transmittance and reflectance spectra of an embedded WGP sheet as a function of *W* for a given value of *H* (500 nm). As can be seen in Fig. 3a, the transmittance for the *y*-polarisation increases when *W* decreases. However, for the *x*-polarisation, the wavelength at which the transmittance goes to zero (light-blocking wavelength) exhibits a red-shift with decreasing values of *W*. Correspondingly, the wavelength at which the reflectance for *x*-polarisation starts to increase exhibits a red-shift, and the total reflectance decreases considerably with decreasing *W*, as can be seen in Fig. 3b. Figure 4 shows the calculated transmittance and reflectance spectra of an embedded WGP sheet as a function of *H* of the wire-grid structure for a given value of *W* (40 nm). The light-blocking performance for the *x*-polarisation undergoes a significant improvement when *H* is increased (Fig. 4a). However, the transmittance for the *y*-polarisation exhibits a decrease with increasing values of *H* within the observed wavelength band as can be seen in Fig. 4b. On the other hand, the reflectance for both *x*- and *y*-polarisations is not significantly affected by *H* as can be seen in Fig. 4c,d. In terms of *H*, we expect based on our calculations, that the polarisation performance of samples fabricated using different moulds to decrease in the following order: Type-C > Type-B > Type-A. On the other hand, in terms of the structural width, we expect that a sample fabricated using Type-D mould to exhibit high transmission values for *y*-polarisation.

**Figure 5:**
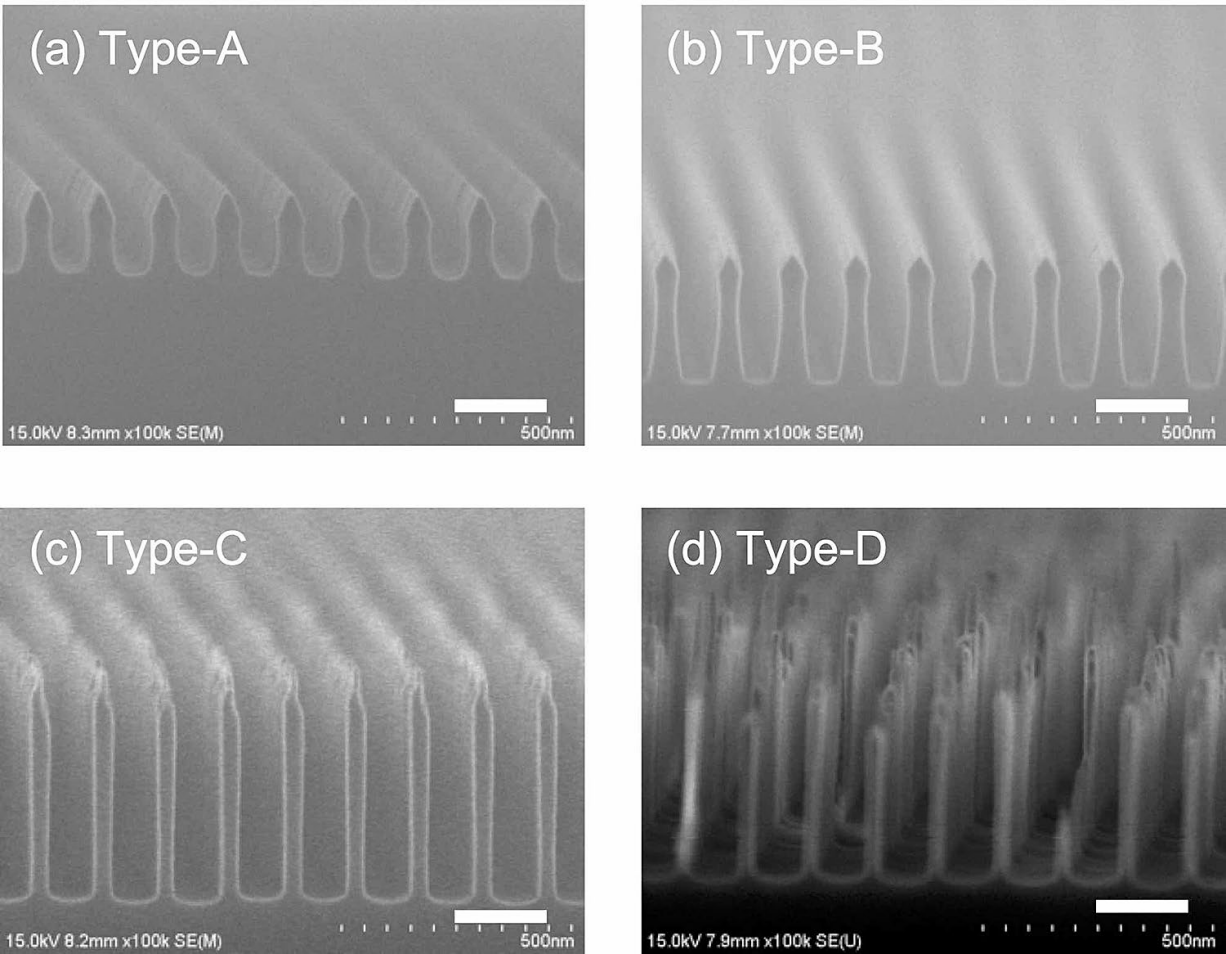
SEM images of the moulds used for the nanoprinting process. The white scale bars in each image represent 200 nm.

Figure 5 shows SEM images of the cross-sections of different moulds. A 4-inch Si wafer was used for their fabrication. We observed that the type-A and type-B moulds had a tapered tip, while the type-C mould had a tapered tip and an uneven surface. The type-D mould had a considerably rougher tip, and the structure exhibited a significant variation in thickness along the wire-direction. These moulds were fabricated by nanoimprint lithography and dry etching processes using a master mould with line-and-space patterns. Depending on the conditions of the nanoimprint lithography and dry etching processes, the width, thickness, and tip shape of the structure can be controlled. Furthermore, the processes can also be adjusted to reduce the roughness of the sidewall.

**Figure 6:**
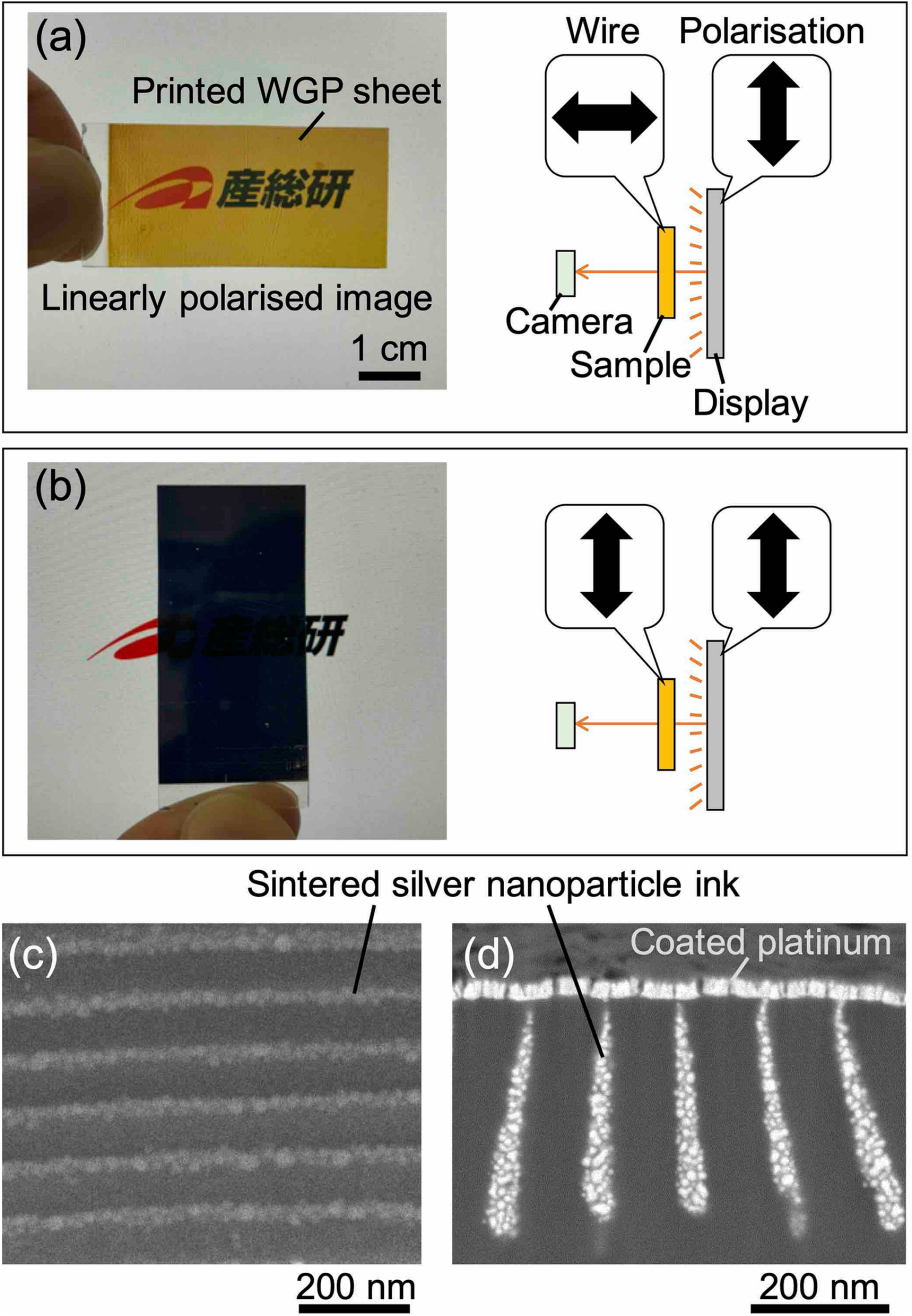
Photographs of the low-reflective WGP sheet over a linearly polarised image. Directions of wires of the WGP sheet are arranged in (**a**) the perpendicular, and (**b**) parallel to the linearly polarised image. (**c**) Top-view and (**d**) cross-sectional-view SEM images of the printed WGP sheet that was fabricated using the type-C mould.

The proposed WGP sheet was fabricated by a nanoprinting process using these moulds. Figure 6a,b show photographs of a WGP sheet fabricated using the type-C mould. As shown in the illustration on the right in these figures, the sample was placed over the linearly polarised image of the LCD. As shown in Fig. 6a, when the sample was placed such that the direction of the nanowire was perpendicular to the polarisation direction of the polarised image, the background image could be seen. On the other hand, as shown in Fig. 6b, when the sample was placed such that the direction of the nanowire was parallel to the polarisation direction of the polarised image, the background image could not be seen. Consequently, the fabricated sample demonstrated its function as a polariser. Figure 6c shows the top view of the SEM image of the fabricated sample. Silver nanoparticles with a diameter of approximately 10–40 nm were densely spread on the top of the periodically formed nanowires. Figure 6d shows the cross-sectional view of the SEM image of the sample. Platinum was coated over the silver nanoparticles for the SEM observation. Due to the thermal deformation caused by the sintering process, leaned nanowires with a high aspect ratio were obtained. The bottom part of the nanowire had a tapered or rounded shape. Observations from this cross-section suggest that there is a variation in thickness between different nanowires, with some wires being approximately 20% lower than others, resulting in an uneven thickness in the direction of the wires. In addition, it is observed that the nanowires formed from the aggregates in which silver particles of several tens of nanometres diameter were partially bonded, and the gap between the particles in the part that did not seem to be bonded was below 10 nm. In this way, high aspect ratio nanowires having irregularities on the surface were realised on both the top and bottom sides of the sample.

**Figure 7:**
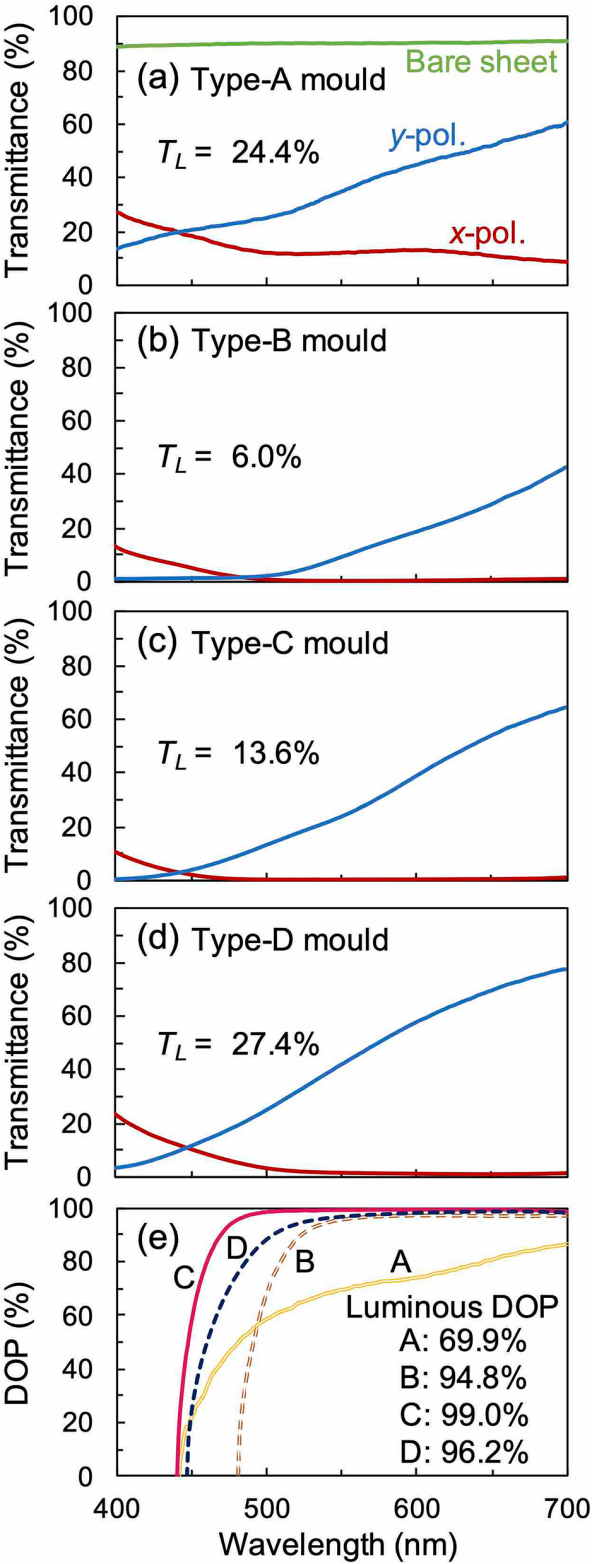
Measured transmittance spectra of WGP sheets printed by using (**a**) type-A, (**b**) type-B, **c** type-C, and (**d**) type-D moulds. (**e**) DOP spectra were obtained from the measured transmission spectra of each sample.

The optical characteristics of a printed WGP sheet were measured using a spectroscope to determine whether it could be used as a polariser in the visible region of the electromagnetic spectrum. Figure 7a–d show the experimental transmittance spectra of WGP sheets fabricated using moulds of type-A, type-B, type-C, and type-D, respectively. The red and blue solid curves represent measurement results when the incident light is polarised in the *x*- and the *y*-direction as it entered each sample, respectively. Figure 7e shows the plot of the degree of polarisation (DOP) with respect to the wavelength of the incident radiation for the WGP sheets fabricated using different moulds. The DOP is a parameter indicating the polarisation performance such as a polarisation extinction ratio, and is represented by the following equation:

2 $${\text{DOP}} = \left\{ {\begin{array}{*{20}c} {\sqrt {\frac{{T_{y} - T_{x} }}{{T_{y} + T_{x} }}} \left( {T_{y} \ge T_{x} } \right)} \\ { - \sqrt {\frac{{T_{x} - T_{y} }}{{T_{x} + T_{y} }}} \left( {T_{y} < T_{x} } \right) } \\ \end{array} } \right..$$

where *T*_*x*_ and *T*_*y*_ represent the transmittances of the samples for the *x*- and the *y*-polarisation, respectively. The threshold wavelength at which the DOP changed from a negative to a positive value was 441, 481, 440, and 447 nm for each of the samples fabricated using the type-A, type-B, type-C, and type-D moulds, respectively. To quantify the performance evaluation, we have introduced the parameters luminous DOP and the luminous transmittance (*T*_*L*_). Luminous DOP was calculated from the measured spectral data and the luminous curve^36^, while *T*_*L*_ was calculated from the spectral data for the total transmittance (*T* = (*T*_*x*_ + *T*_*y*_)/2) and the spectral data of the luminous curve.

Polarisation anisotropy was observed for samples fabricated using the type-A mould. A transmittance of 12.2% was obtained at a wavelength of 550 nm for the *x*-polarisation (i.e., along the light-blocking axis). This value indicates a weak light-blocking performance as a polariser. At the same wavelength, a transmittance of 35.0% was obtained for the *y*-polarisation. The calculated DOP for this sample was 69.4% and the luminous DOP was also quite low. Consequently, the samples fabricated using the type-A mould are not good for our desired polarising applications. Based on the results obtained by the RCWA calculations (in Fig. 4), the low value of luminous DOP can be attributed to the relatively low values of the optical thickness of the nanowires due to the low *H* of the mould.

The light-blocking performance for the sample fabricated using the type-B mould improved considerably, particularly for the *x*-polarised light, and transmittance of less than 1% was obtained for wavelengths longer than 497 nm. The transmittance reached 0.38% at a wavelength of 550 nm. In contrast, for *y*-polarisation, the transmittance was in general lower than that observed for the type-A mould within this wavelength band. It was 9.4% at a wavelength of 550 nm and 42.6% at a wavelength of 700 nm. However, the DOP exceeded 90% at a wavelength of 523 nm, reaching 96.0% at a wavelength of 550 nm. The luminous DOP increased to 94.8%. This increase can be understood in terms of the increased optical thickness of the printed nanowires (Fig. 4) due to the higher value of *H* for this mould.

For the samples fabricated using the type-C mould, we observed that the light-blocking performance for *x*-polarised light was better than the sample fabricated using the type-B mould. Transmittance of less than 1% was observed for wavelengths longer than 465 nm, which further dropped to a value of 0.22% at a wavelength of 550 nm. The transmittance for the *y*-polarisation also showed an increase when compared with the sample fabricated using the type-B mould. The transmittance was 23.9% at a wavelength of 550 nm and 64.3% at a wavelength of 700 nm. The DOP exceeded 90% at a wavelength of 470 nm, reaching 99.1% at a wavelength of 550 nm. The luminous DOP was 99.0% because of the high value of *H* for this mould which is approximately 1.8 times higher compared to the type-B mould. The luminous DOP obtained, in this case, satisfies the polarisation characteristics of commercially available polarised sunglasses, where it is mandatory to have at least a luminous DOP of 90%. However, a luminous DOP of 99.0% or higher is often used as a standard in the current Japanese market^37^. The experimental transmittance spectra obtained for the samples fabricated using type-C mould are in good agreement with the spectra obtained from simulation (Fig. 2a,b). This can be explained by considering that the conductivity of the sintered body of silver nanoparticles is lower than that of bulk silver.

In the case of samples fabricated using the type-D mould, the light-blocking performance for the *x*-polarised light slightly weakened. Transmittances of just 1.3% were observed at a wavelength of 550 nm which decreased to a value of less than 1% for wavelengths longer than 587 nm. In contrast, the transmittance for the y-polarisation was significantly enhanced compared to the type-C mould, being 41.8% at a wavelength of 550 nm and reaching 77.4% at a wavelength of 700 nm. The DOP exceeded 90% at a wavelength of 504 nm, reaching 97.0% at a wavelength of 550 nm. Comparing the experimental results with the simulation (Fig. 3), it was confirmed that the *T*_*L*_ increased to 27.4% because of the narrowing of *W* to a value which is about half that of the other moulds.

The line width, pitch, and material of the nanowires were the primary reasons why the printed WGP sheets exhibited weak polarisation characteristics in the shorter wavelength region (400 nm). In the case of silver, the real part of permittivity approaches zero from the blue to the ultraviolet region^38^. As a result, the metallic behaviour of silver diminishes in the shorter wavelength region which could lead to an increase in the transmittance for the *x*-polarisation in this region. The decrease in transmittance for the *y*-polarisation in the shorter wavelength region could be attributed to the surface plasmon resonance within the wires^39^. It is plausible that the plasmon absorption due to the unsintered (or partially sintered) silver nanoparticles present in the WGP sheet affects the reduction of the transmittance at *y*-polarisation in the shorter wavelength region When comparing the WGP sheets fabricated using the type-B and type-D moulds, the wavelength at which the transmittance of *y*-polarisation start to increase was shorter than when the type-D mould was used. This is because the width to pitch ratio of the nanowires was small (Fig. 3). Therefore, by increasing the conductivity of nanowires as well as by optimising their shape, the WGP sheet made of silver nanoparticle ink can function as a polariser in the shorter wavelength region.

Furthermore, polarisation performance was measured as a function of the incidence angle, which was varied from 0 to 70° in 5° increments in each of the *x*–*z* and *y*–*z* planes. The maximum change of the measured luminous DOP equalled 0.2%, indicating robust polarisation performance with respect to the incidence angle.

**Figure 8:**
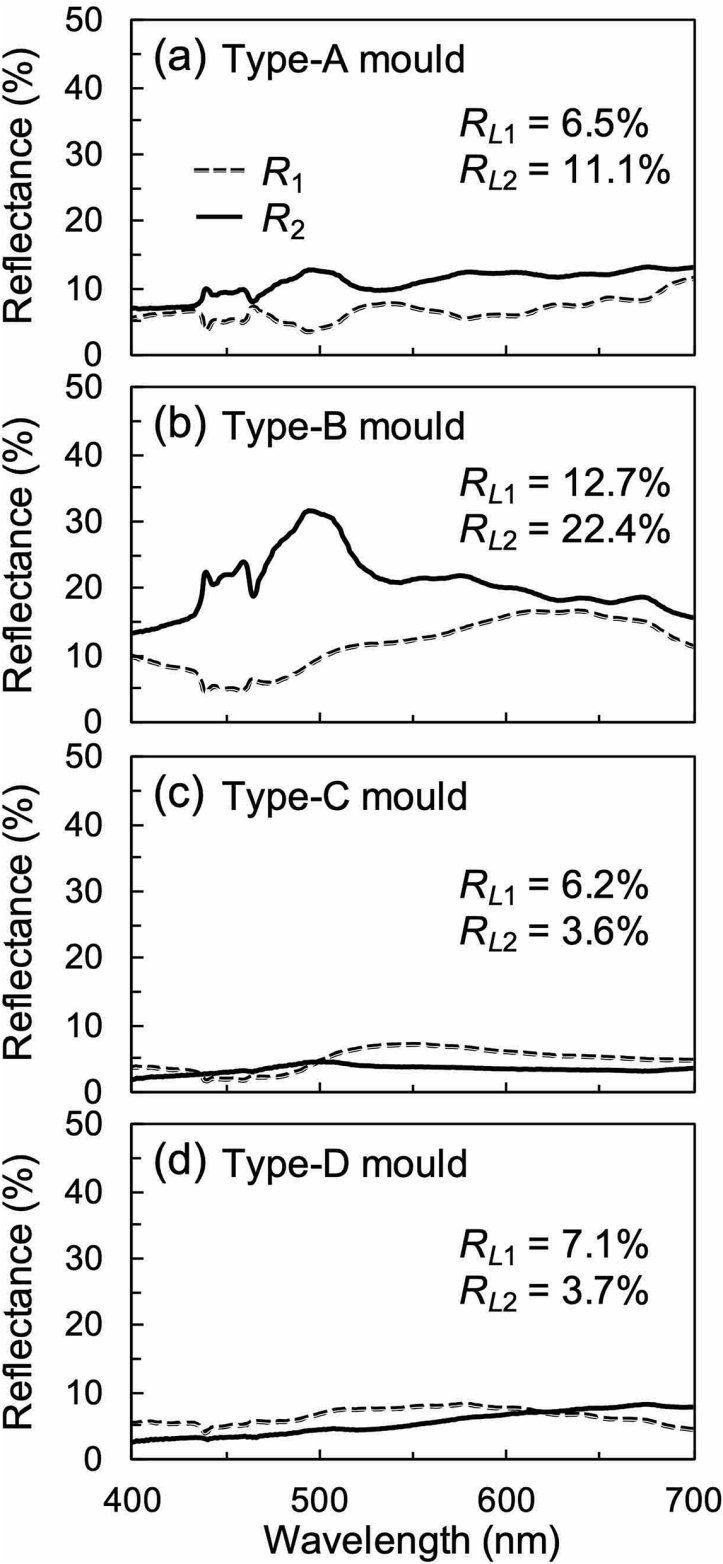
Measured reflectance spectra of WGP sheets printed by using (**a**) type-A, (**b**) type-B, **c** type-C and **d** type-D moulds. The incident light is unpolarised.

**Figure 9:**
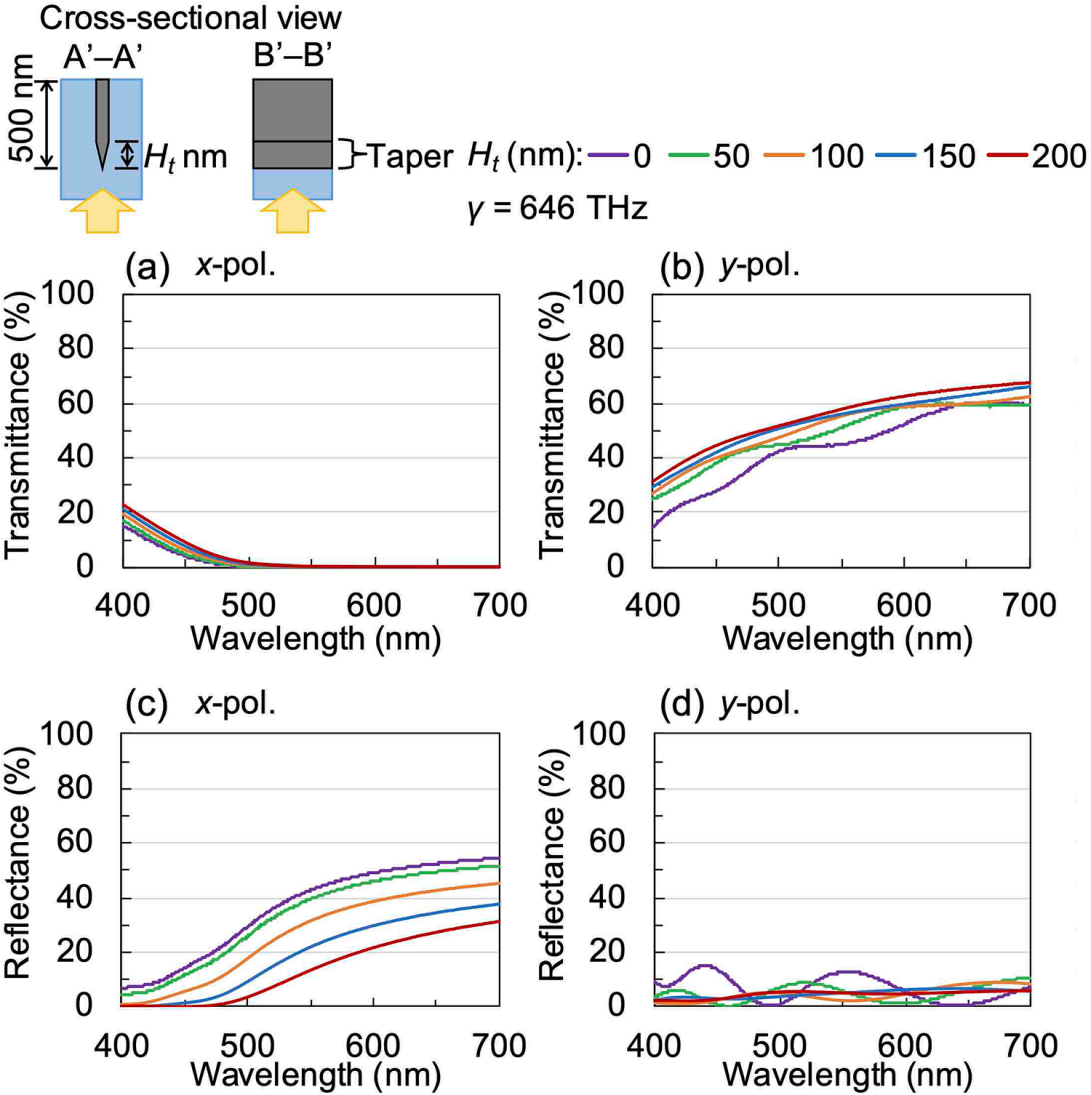
Calculated **a**, **b** transmittance and **c**, **d** reflectance spectra of embedded WGP sheet with a tapered tip as a function of *H*_*t*_. The polarisation directions are **a**, **c** *x*-polarisation and **b**, **d** *y*-polarisation.

**Figure 10:**
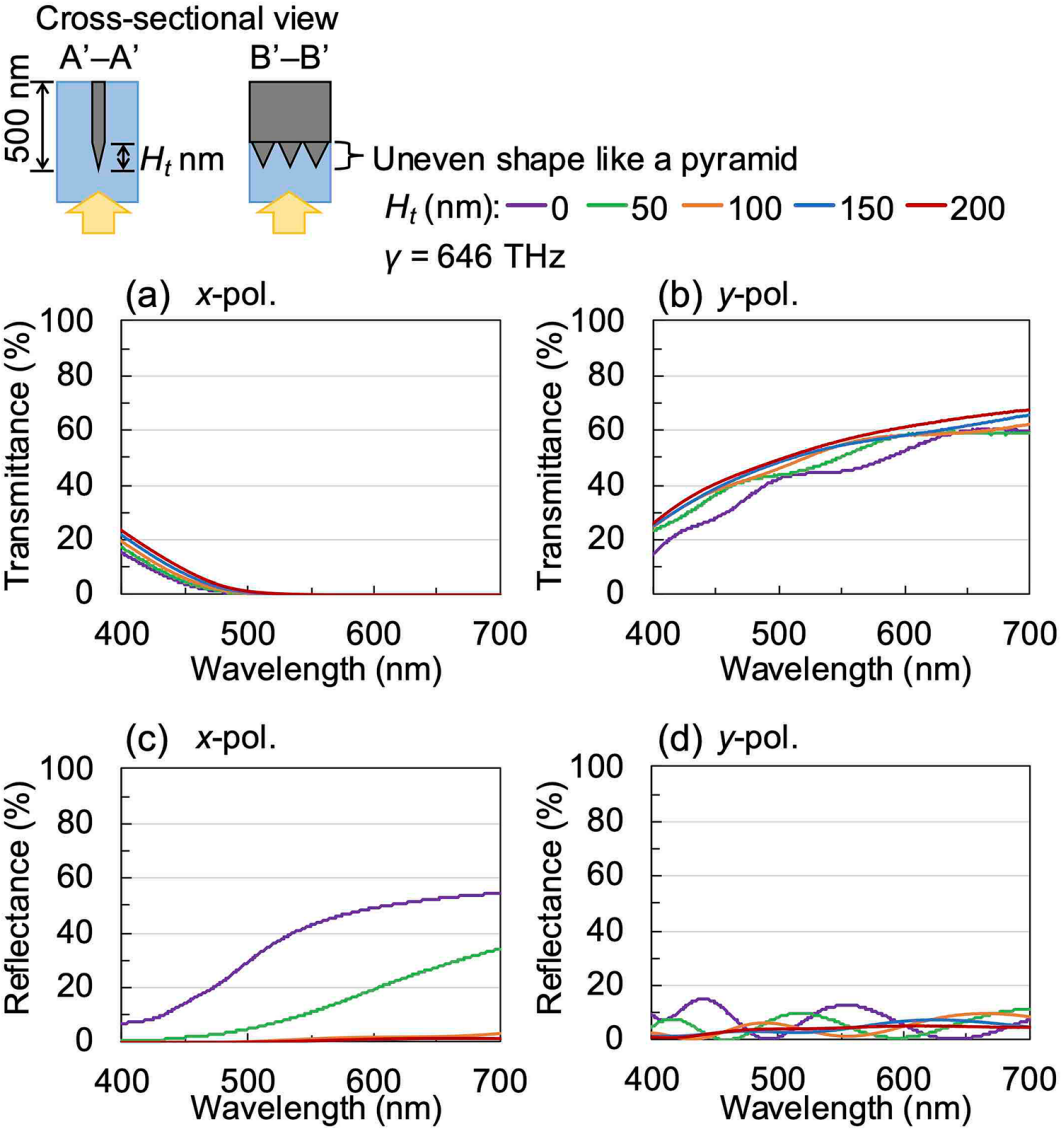
Calculated **a**, **b** transmittance and **c**, **d** reflectance spectra of embedded WGP sheet with an uneven shape like a pyramid as a function of *H*_*t*_. The polarisation directions are **a**, **c** *x*-polarisation and **b**, **d** *y*-polarisation.

Using microspectrophotometer, we have measured the reflectance spectra of the printed WGP sheets fabricated using the respective moulds as can be seen in Fig. 8a–d. These measurements were carried out using unpolarised incident light. *R*_1_ (shown by a broken line) represents the reflectance obtained from the top of the printing surface, and *R*_2_ (shown by a solid line) represents the reflectance from the bottom of the nanowires from the back. When the printed WGP sheets were applied to sunglasses, a low value of *R*_2_ was required because the back of the nanowires was arranged within the lens. The luminous reflectance (*R*_*L*1_, *R*_*L*2_) was calculated from the measured spectral data and the luminous curve. Compared to the WGPs fabricated using conventional methods^15,16^, the reflectance spectra of the WGP samples fabricated using silver nanoparticle ink showed low reflectance within the visible region of the electromagnetic spectrum. When viewed in conjunction with the transmittance spectra of Fig. 7, especially for the samples fabricated using the type-C and type-D moulds, the reflectance did not increase significantly and exhibited a small value (less than 10%) even in the longer wavelength region. To demonstrate the effect of the wire-grid structure with a tapered tip or uneven surface we have calculated its optical characteristics. In these calculations, *W*, *H*, and *γ* were set to 40 nm, 500 nm, and 646 THz, respectively. The total thickness *H* includes the thickness of the tapered portion *H*_*t*_. Figure 9 shows the calculated transmittance and reflectance spectra of embedded WGP sheets with a tapered tip as a function of *H*_*t*_ which was varied from 0 to 200 nm. The tapered portion was designed to linearly narrow down toward the tip, and the layer was divided for each *H*_*t*_/10 for the calculations. It seems that the tapered tip provides slightly higher transmittance than the flat tip as shown in Fig. 9a,b. Importantly, it was confirmed that the reflectance was reduced with an increase of *H*_*t*_ especially for *x*-polarised light, and the reflectance decreases to 14% at a wavelength of 550 nm and *H*_*t*_ of 200 nm as shown in Fig. 9c,d. Figure 10 shows the calculated transmittance and reflectance spectra of an embedded WGP sheet with an uneven shape (like a pyramid) as a function of *H*_*t*_ which was varied from 0 to 200 nm. The tip structure had a shape that linearly narrows toward the tip with a bottom dimension of 50 and 40 nm in the *x*- and *y*-direction, respectively, and was arranged in 50 nm cycles in the *x*-direction. In this calculation, the layer was also divided for each *H*_*t*_/10. Compared with the calculation result for the tapered tip, the change in the transmittance was comparable as shown in Fig. 10a, b. Tip structure with the uneven shape proved to be more effective in reducing the reflectance compared to the tapered shape, and the calculated reflectance was close to zero at *H*_*t*_ ~ 100 nm as shown in Fig. 10c, d. From the SEM images, the tip of the structure of the type-A and type-B moulds can be considered as tapered, while the tip of the type-C and type-D mould can be considered uneven (see Fig. 5). *R*_2_ for the WGP sheet fabricated using the type-A and type-B moulds with *H*_*t*_ of the tapered shape equalled ~ 50 nm or 40 nm, as shown in Fig. 9c. It is presumed that these tip shapes contribute negligibly towards the reduction in reflectance. On the other hand, for the WGP sheet fabricated using the type-C and type-D moulds with *H*_*t*_ of the uneven shape of 100 nm or 180 nm, exhibit excellent reflectance reduction which can be attributed to the tip shape as can be seen from Fig. 10c. Experimentally, low luminous reflectance of 3.6% and 3.7% were obtained. Similarly for *R*_1_, the result was affected by the nature of the surface (i.e., presence of sub-wavelength surface roughness) in addition to the optical absorption by the sintered body of silver nanoparticle ink with low conductivity. As described above, the reflectance can be significantly reduced compared to the conventional WGP by using the sintered body of silver nanoparticle ink and controlling the tip shape.

**Figure 11:**
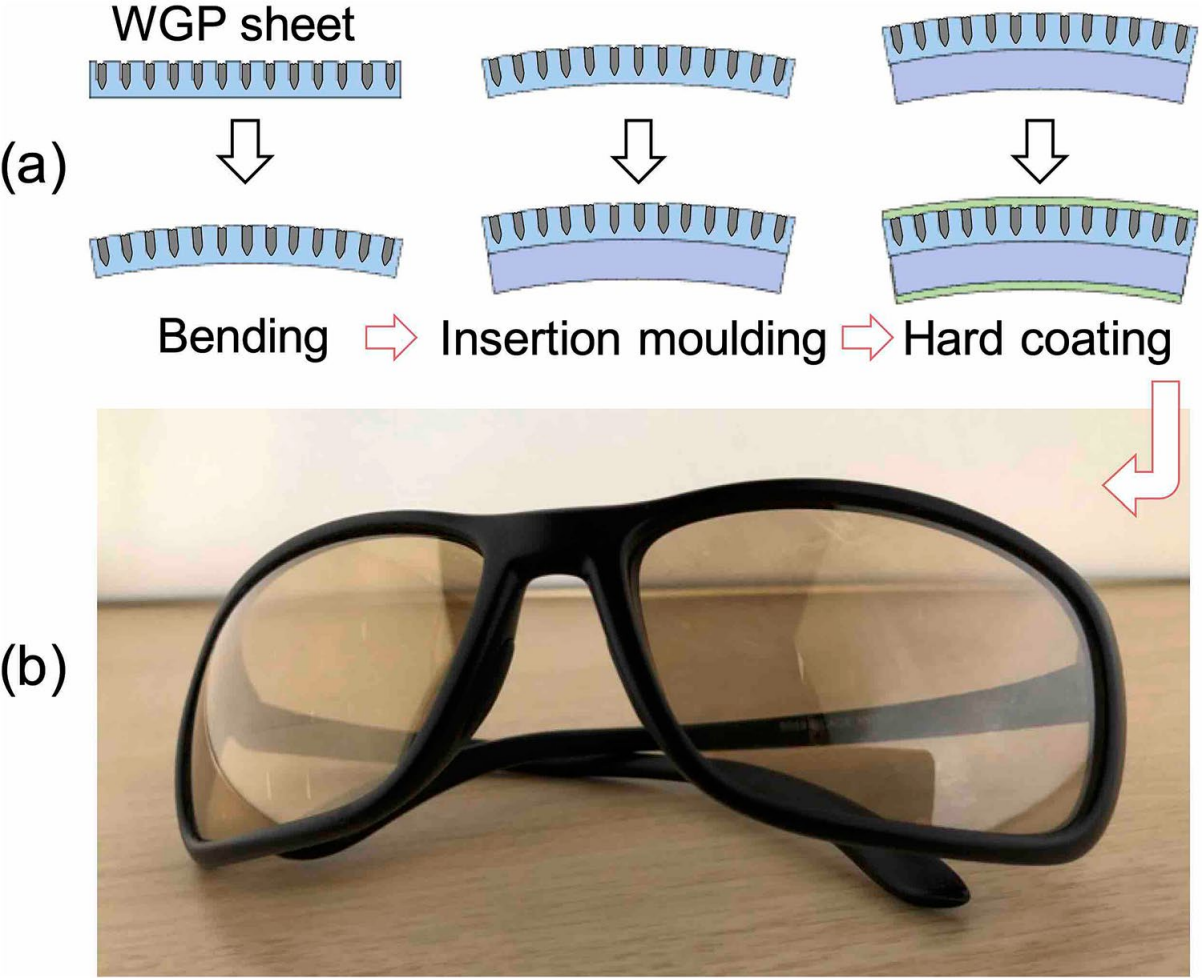
**a** A process flow for polarised sunglasses. **b** Photograph of the polarised sunglasses using the printed low-reflective WGP sheet.

Finally, we report the trial production of polarised sunglasses using the printed WGP sheet. Figure 11a schematically represents the different steps such as the bending step, insertion moulding step, and hard-coating step, involved in the process. In the bending step, the surface of the sheet was strained by bending stress, but no damage to the printed nanowires was observed. In the insertion moulding step, even though the printed WGP sheet was exposed to a high-temperature resin, this did not affect its optical characteristics. In the hard-coating step, no deformation or damage to the printed nanowires was observed. Figure 11b shows a prototype of polarised sunglasses. We succeeded in fabricating it at the size of actual sunglasses and with an appearance comparable to that of a commercially available product. We used a printed WGP sheet with a luminous DOP of 98.9% and luminous transmittance of 10.8%. The optical characteristics of the polarised sunglasses after the bending, insertion moulding, and hard coating steps, showed a luminous DOP of 98.9% and luminous transmittance of 13.5%. Since there was almost no effect on the luminous DOP, we conclude that the printed nanowires themselves did not undergo any large deformation which might have reflected as a change in their optical properties. In addition, there was almost no change in reflectance. Therefore, the printed WGP sheet exhibited the durability of sustaining the manufacturing process for curved products including sunglasses.

## Conclusions

We have fabricated a low-reflectivity WGP sheet for the visible portion of the electromagnetic spectrum by the nanoprinting process using silver nanoparticle ink. Compared to the conventional nanofabrication techniques for a WGP, the proposed method makes it easier to achieve multiple wafer-scale productions without using sophisticated equipment. The optical characteristics of the WGP sheet can be tuned by controlling the shape of the printed nanowires, such as providing irregularities on the mould tip and the surface of the nanowires. A WGP sheet with a luminous DOP of 99.0% and a luminous reflectance of 3.6% was fabricated which was durable enough to withstand the sunglass moulding process. The fabrication of optical structures by the proposed nanoprinting process using nanoparticle ink has the potential to significantly contribute to the development of fine-structured optical elements with unprecedented functionality.

## Methods

*Fabrication of WGP sheets*: First, the moulds were fabricated by nanoimprint lithography and reactive ion etching (RIE) on a 4-inch Si wafer. The shape (width, thickness, and tip thickness) of the inverted wire-grid structures was controlled using the RIE conditions. For the substrate, a 400-µm thick PC sheet (FE-2000, Mitsubishi Gas Chemical) was employed. Wire-grid grooves were formed on a substrate sheet by using a thermal nanoimprint (hot embossing) process, which was carried out in a vacuum. The imprint pressure, temperature, and time were 0.9 MPa, 175 °C, and 150 s, respectively. Subsequently, the nanogrooves were filled with silver nanoparticle ink (NPS, Harima Chemicals), having a representative primary particle diameter of 12 nm and metal content of 83%, using a squeegeeing method. Following this process, some ink remained outside the nanogrooves. The samples were wiped to remove this excess ink. Finally, sintering was performed at 130 °C using an oven. The sintered bodies of nanoparticle ink were formed, which became silver wire-grid structures with irregularities on the surface.

*Measurements* Transmittance spectra were measured using a spectroscope (SolidSpec-3700, Shimadzu). The measurement area was 2 mm square. Reflectance spectra were measured using a confocal microspectrometer (USPM-RU III, Olympus). SEM images of the moulds and a top view of the fabricated WGP sheets were obtained using a field emission SEM (S-4800, Hitachi). Cross-sectional images of the fabricated WGP sheets were obtained using a hybrid ion milling system (IM4000Plus, Hitachi) and field-emission SEM (Regulus8240, Hitachi).

*Simulations* Numerical simulations were carried out using the 3D optical diffraction simulator (DiffractMOD, Cybernet).

## References

[1] Kim SH, Park J-D, Lee K-D (2006). Fabrication of a nano-wire grid polarizer for brightness enhancement in liquid crystal display. Nanotechnology.

[2] Weber, M. F., Ouderkirk, A. J., Wheatley, J. A. and Brodd, J. (3M) *US6952312B2* 2002.

[3] Dirix Y, Tervoort TA, Bastiaansen C (1995). Optical properties of oriented polymer/dye polarizers. Macromolecules.

[4] Peeters E, Lub J, Steenbakkers JAM, Broer DJ (2006). High-contrast thin-film polarizers by photo-crosslinking of smectic guest-host systems. Adv. Mater..

[5] Ekinci Y, Solak HH, David C, Sigg H (2006). Bilayer Al wire-grids as broadband and high-performance polarizers. Opt. Express.

[6] Yang ZY, Lu YF (2007). Broadband nanowire-grid polarizers in ultraviolet-visible-near-infrared regions. Opt. Express.

[7] Takano K (2010). Fabrication of terahertz planar metamaterials using a super-fine ink-jet printer. Appl. Phys. Express.

[8] Ferraro A (2016). Flexible terahertz wire grid polarizer with high extinction ratio and low loss. Opt. Lett..

[9] Shin YJ, Wu Y-K, Lee K-T, Ok JG, Guo LJ (2013). Fabrication and encapsulation of a short-period wire grid polarizer with improved viewing angle by the angled-evaporation method. Adv. Opt. Mater..

[10] Xu M, Urbach HP, de Boer DKG, Cornelissen HJ (2005). Wire-grid diffraction gratings used as polarizing beam splitter for visible light and applied in liquid crystal on silicon. Opt. Express.

[11] Chen L (2007). Large flexible nanowire grid visible polarizer made by nanoimprint lithography. Appl. Phys. Lett..

[12] Ahn SH, Kim J-S, Guo LJ (2007). Bilayer metal wire-grid polarizer fabricated by roll-to-roll nanoimprint lithography on flexible plastic substrate. J. Vac. Sci. Technol. B.

[13] Takano K, Yokoyama H, Ichii A, Morimoto I, Hangyo M (2011). Wire-grid polarizer sheet in the terahertz region fabricated by nanoimprint technology. Opt. Lett..

[14] Kwon S, Lu D, Sun Z, Xiang J, Liu Z (2016). Highly stretchable, printable nanowire array optical polarizers. Nanoscale.

[15] Suzuki M (2010). Low-reflective wire-grid polarizers with absorptive interference overlayers. Nanotechnology.

[16] Lee JH (2008). Optically bifacial thin-film wire-grid polarizers with nano-patterns of a graded metal-dielectric composite layer. Opt. Express.

[17] Suzuki M (2011). Antireflection coatings with FeSi_2_ layer: Application to low-reflectivity wire grid polarizers. Thin Solid Films.

[18] Yamada I (2009). Transmittance enhancement of a wire-grid polarizer by antireflection coating. Appl. Opt..

[19] Kim JH, Cho YT, Jung YG (2016). Selection of absorptive materials for non-reflective wire grid polarizers. Int. J. Precis. Eng. Manuf..

[20] Wilson SJ, Hutley MC (1982). The optical properties of 'moth eye' antireflection surfaces. Opt. Acta.

[21] Ji S, Park J, Lim H (2012). Improved antireflection properties of moth eye mimicking nanopillars on transparent glass: Flat antireflection and color tuning. Nanoscale.

[22] Leem JW (2014). Efficiency enhancement of organic solar cells using hydrophobic antireflective inverted moth-eye nanopatterned PDMS films. Adv. Energy Mater..

[23] Sun C-H, Jiang P, Jiang B (2008). Broadband moth-eye antireflection coatings on silicon. Appl. Phys. Lett..

[24] Chen Q (2009). Broadband moth-eye antireflection coatings fabricated by low-cost nanoimprinting. Appl. Phys. Lett..

[25] Yang J (2014). Design and fabrication of broadband ultralow reflectivity black Si surfaces by laser micro/nanoprocessing. Light Sci. Appl..

[26] Christiansen AB (2015). Black metal thin films by deposition on dielectric antireflective moth-eye nanostructures. Sci. Rep..

[27] Tan X, Tao Z, Yu M, Wu H, Li H (2018). Anti-reflectance investigation of a micro-nano hybrid structure fabricated by dry/wet etching methods. Sci. Rep..

[28] Kim H-S, Dhage SR, Shim D-E, Hahn HT (2009). Intense pulsed light sintering of copper nanoink for printed electronics. Appl. Phys. A.

[29] Hokari R, Kurihara K, Takada N, Hiroshima H (2017). Development of simple high-resolution embedded printing for transparent metal grid conductors. Appl. Phys. Lett..

[30] Hokari R, Kurihara K, Takada N, Hiroshima H (2018). Printed optical metamaterials composed of embedded silver nanoparticles for flexible applications. Opt. Express.

[31] Hokari R, Kurihara K, Higurashi E, Hiroshima H (2019). Optical evaluation of nanocomposite metamaterials fabricated by nano-printing technique utilizing silver nanoink. Microelectron. Eng..

[32] Moharam MG, Gaylord TK (1986). Rigorous coupled-wave analysis of metallic surface-relief gratings. J. Opt. Soc. Am. A.

[33] Ishikawa A, Tanaka T, Kawata S (2005). Negative magnetic permeability in the visible light region. Phys. Rev. Lett..

[34] Johnson PB, Christy RW (1972). Optical constants of the noble metals. Phys. Rev. B.

[35] Chung W-H, Hwang H-J, Lee S-H, Kim H-S (2013). In situ monitoring of a flash light sintering process using silver nano-ink for producing flexible electronics. Nanotechnology.

[36] Rakić AD, Djurišić AB, Elazar JM, Majewski ML (1998). Optical properties of metallic films for vertical-cavity optoelectronic devices. Appl. Opt..

[37] Consumer Affairs Agency, Government of Japan, https://www.caa.go.jp/policies/policy/representation/household_goods/guide/zakka/zakka_24.html. Accessed 21 August 2020 **(Japanese)**.

[38] ISO 8980-3:2013, Ophthalmic optics—Uncut finished spectacle lenses—Part 3: Transmittance specifications and test methods.

[39] Wiley BJ, Im SH, Li Z-Y, McLellan J, Siekkinen A, Xia Y (2006). Manoeuvring the surface plasmon resonance of silver nanostructures through shape-controlled synthesis. J. Phys. Chem. B.

